# Reduced Ceftazidime-Avibactam Susceptibility in KPC-Producing *Klebsiella pneumoniae* From Patients Without Ceftazidime-Avibactam Use History – A Multicenter Study in China

**DOI:** 10.3389/fmicb.2020.01365

**Published:** 2020-06-23

**Authors:** Xiaoyan Cui, Bin Shan, Xue Zhang, Fen Qu, Wei Jia, Bin Huang, Hua Yu, Yi-Wei Tang, Liang Chen, Hong Du

**Affiliations:** ^1^Department of Clinical Laboratory, The Second Affiliated Hospital of Soochow University, Suzhou, Jiangsu, China; ^2^Department of Clinical Laboratory, The First Affiliated Hospital of Kunming Medical University, Kunming, China; ^3^Center for Clinical Laboratory, 302 Hospital of PLA, Beijing, China; ^4^Aviation General Hospital, China Medical University, Beijing, China; ^5^Medical Laboratory Center, General Hospital of Ningxia Medical University, Yinchuan, China; ^6^Department of Laboratory Medicine, The First Affiliated Hospital, Sun Yat-sen University, Guangzhou, China; ^7^Sichuan Academy of Medical Sciences Sichuan Provincial People’s Hospital, Chengdu, China; ^8^Department of Laboratory Medicine, Memorial Sloan Kettering Cancer Center, New York, NY, United States; ^9^Department of Pathology and Laboratory Medicine, Weill Cornell Medicine, Cornell University, New York, NY, United States; ^10^Cepheid, Shanghai, China; ^11^Center for Discovery and Innovation, Hackensack Meridian Health, Nutley, NJ, United States; ^12^Hackensack Meridian School of Medicine at Seton Hall University, Nutley, NJ, United States

**Keywords:** KPC, *Klebsiella pneumoniae*, ceftazidime-avibactam, antimicrobial susceptibility, antimicrobial resistance

## Abstract

*KPC-producing Klebsiella pneumoniae* (KPC-KP) is the most widely spread carbapenem-resistant *Enterobacteriaceae* (CRE) in China. Avibactam is a novel non-β-lactam β-lactamase inhibitor which is highly active against KPC. Recently, *ceftazidime-avibactam* (CAZ-AVI) was approved for clinical treatment in China. Here we conducted a retrospective study to examine the antimicrobial susceptibility of CAZ-AVI prior to its usage in China, and evaluated the potential to develop resistance in KPC-KP. CAZ-AVI MICs were tested in 347 KPC-KP isolates collected from patients with no prior treatment with this combination from six medical centers in China. Almost all isolates (*n* = 346; 99.7%) were CAZ-AVI-susceptible, with only 12 (3.5%) which showed reduced susceptibility (MIC ≥ 4/4 μg/ml) or resistance. The 12 isolates belong to ST11 and half of them carry virulence genes. In comparison to susceptible isolates, these isolates demonstrated higher *bla*_*KPC–2*_ copy numbers and expressions, and demonstrated higher frequency of developing CAZ-AVI resistance.

## Introduction

With the widely use of carbapenem antibiotics, carbapenem-resistant *Enterobacteriaceae* (CRE) have been increasingly detected worldwide. The production of carbapenemases is the leading cause of carbapenem resistance in CRE. *Klebsiella pneumoniae* carbapenemase (KPC) is currently the most widely spread carbapenemase in the world, including China (Nordmann and Poirel, 2014), while *K. pneumoniae* is the main clinical species producing KPC (Zhang et al., 2017). KPC-type β-lactamases could hydrolyze carbapenems and almost all β-lactam antibiotics, and traditional β-lactamase inhibitors have limited effects on KPC (Papp-Wallace et al., 2010). In addition, isolates producing KPC are commonly resistant to many other clinical agents (Pollett et al., 2014) due to the co-expression of several resistant determinants. Novel treatments for infections caused by KPC-producing *K. pneumoniae* (KPC-KP) are in urgent need.

Avibactam, a novel non-β-lactam β-lactamase inhibitor, had a spectrum of activity against β-lactamase of classes A (e.g., KPC), C (AmpC), and selected D (e.g., OXA-48) enzymes (van Duin and Bonomo, 2016). The combination of ceftazidime-avibactam (*CAZ-AVI)* has been approved in clinical treatment for KPC-producing *Enterobacteriaceae* by the United States Food and Drug Administration (FDA) in 2015 (FDA, 2015). In China, injectable CAZ-AVI (ZAVICEFTA^®^) was recently (May 2019) approved for the treatment of complicated intra-abdominal infections (cIAI) and hospital-acquired pneumonia (HAP), including ventilator associated pneumonia (VAP), caused by multidrug resistant Gram-negative bacteria.

In this study, we examined the CAZ-AVI susceptibility of KPC-KP from patients with no prior CAZ-AVI treatment history in China and evaluated the potential of isolates with reduced susceptibility to this combination to develop resistance through *in vitro* selection experiments.

## Materials and Methods

### Collection and Identification of Carbapenem-Resistant *K. pneumoniae*

A total of 616 unique clinical *K. pneumoniae* displaying carbapenem resistance which were defined as any isolate displaying imipenem and/or meropenem MIC values of >2 μg/ml based on CLSI guideline (Clinical and Laboratory Standards Institute, 2018) were collected from September 2016 to June 2018 from six tertiary hospitals in six different cities in China including Chengdu (Southwest China), Kunming (Southwest China), Guangzhou (Southern China), Yinchuan (Northwest China), Suzhou (Eastern China), and Beijing (Northern China). Species identification was performed by matrix-assisted laser desorption ionization-time of flight mass spectrometry (MALDI-TOF MS) method using the Bruker Daltonics MALDI Biotyper according to the instructions of manufacturer, and confirmed by 16S rRNA sequencing (Weisburg et al., 1991).

### Detection of Carbapenemases, ESBLs and Analysis of Porin Encoding Genes

Polymerase chain reaction (PCR) was performed to detect the presence of carbapenemase-encoding genes (*bla*_*KPC*_, *bla*_*NDM*_, *bla*_*IMP*_, *bla*_*VIM*_, *bla*_*OXA–48*_) (Candan and Aksoz, 2015), the extended-spectrum beta-lactamases (ESBLs) genes (*bla*_*CTX–M*_, *bla*_*SHV*_ and *bla*_*TEM*_) (Bokaeian et al., 2015) and to amplify porin encoding genes (*ompK35* and *ompK36*) (Clancy et al., 2013) in the carbapenem-resistant *K. pneumoniae*. The entire coding sequences for these genes were then amplified using previously published primers and conditions (Costa et al., 2009; Schechner et al., 2009) and subjected to Sanger sequencing.

### Determination of CAZ-AVI Minimal Inhibitory Concentrations

The CAZ-AVI minimal inhibitory concentrations (MICs) were determined using the broth microdilution method recommended by CLSI (Clinical and Laboratory Standards Institute, 2018). *Escherichia coli* ATCC 25922 was used as quality control strain (Clinical and Laboratory Standards Institute, 2018). MICs were interpreted according to CLSI breakpoints (Clinical and Laboratory Standards Institute, 2018). A CAZ-AVI MIC of ≥4/4 μg/ml was used as the cut-off of reduced susceptibility to CAZ-AVI for this study (Shen et al., 2017).

### Antimicrobial Susceptibility Testing

*In vitro* susceptibility of the isolates with reduced CAZ-AVI susceptibility against the commonly used clinical antimicrobials was evaluated by Phoenix 100 Automated Microbiology System and interpreted using CLSI (Clinical and Laboratory Standards Institute, 2018) guidelines or European Committee on Antimicrobial Susceptibility Testing (EUCAST) (for moxifloxacin and colistin). A total of 20 antibiotics belonging to 11 classes of antimicrobials were tested, including carbapenems (imipenem and meropenem), ureidopenicillin (piperacillin), β-lactam/β-lactamase inhibitor complexes (amoxicillin-clavulanate, ampicillin-sulbactam, and piperacillin-tazobactam), cephalosporins (cefazolin, cefotaxime, ceftazidime, and cefepime), monocyclic β-lactams (aztreonam), aminoglycosides (gentamicin and amikacin), fluoroquinolones (ciprofloxacin, moxifloxacin and levofloxacin), folate metabolic pathway inhibitors (trimethoprim-sulfamethoxazole), chloramphenicol, colistin, and tetracycline.

### Collection of Clinical Information

The clinical information including city, age range, isolation date, clinical department, sample, medical condition and outcome of the patients from whom the carbapenem-resistant *K. pneumoniae* was isolated was collected using EPIINFO software based on the medical records.

### Multilocus Sequence Typing (MLST)

Multilocus sequence typing (MLST) was conducted to investigate the genetic relationships of the isolates with reduced CAZ-AVI susceptibility. PCR followed by Sanger sequencing was used to detect conserved housekeeping including *gapA*, *infB*, *mdh*, *pgi*, *phoE*, *rpoB*, and *tonB* (Diancourt et al., 2005). Allelic profiles and sequence types (STs) were determined using the *K. pneumoniae* MLST database^1^.

### Pulsed-Field Gel Electrophoresis (PFGE)

The clonal relatedness between the isolates with reduced CAZ-AVI susceptibility was investigated by pulsed-field gel electrophoresis (PFGE) with XbaI-digested DNA using a CHEF Mapper Power Module instrument (Bio-Rad, United States). Conditions of electrophoresis were as follow: voltage 6V/cm, running time 20 h, temperature 14°C, and pulse times of 1–40 s. *Salmonella enterica* serotype Braenderup H9812 was used as size marker. The results were evaluated using GelJ v.2.0 analyzing software.

### Investigation of the Capsular Types and Virulence Genes

Multiplex PCR-II analysis was applied to investigate the capsular types including K1, K2, KL64, KL47 (Yu et al., 2018), and multiplex PCR-III analysis was performed to detect four virulence genes previously described on the pLVPK virulence plasmid namely *rmpA*, *rmpA2*, *iroN*, and *iutA* (Yu et al., 2018) in the isolates with reduced CAZ-AVI susceptibility.

### Quantitative Real-Time PCR (qRT-PCR) and *bla*_*KPC*_ Promoter Region Sequencing

A total of 16 isolates, including 8 isolates with reduced susceptibility and 8 randomly selected susceptible isolates, were examined by quantitative real-time PCR (qRT-PCR) to assess the *bla*_*KPC–2*_ copy numbers relative to an internal *K. pneumoniae* housekeeping gene, *rpoB*, as previously described (Kitchel et al., 2010). The expression of *bla*_*KPC–2*_ in the same isolate set was measured using DNA-free RNA preparations by qRT-PCR as described previously (Ruzin et al., 2005; Doumith et al., 2009). The statistical software used in this study was Prism 5 (Graph Pad Software). In addition, the *bla*_*KPC*_ promoter regions from 12 isolates with reduced CAZ-AVI susceptibility and the 8 randomly selected susceptible isolates were amplified and sequenced using the primers (KPCpro-F, 5′-AACGGTCGTATCAGCGACAT-3′ and KPCpro-R, 5′-CGAGTTTAGCGAATGGTTCC-3′), covering the previously described promoter regions in KPC-KP isolates from China (Huang et al., 2019).

### *In vitro* Selection Testing

All isolates underwent qRT-PCR detection, except for the CAZ-AVI (MIC 16/4 μg/ml) resistant isolate (*n* = 15), were subject to *in vitro* CAZ-AVI selective pressure testing, using a previously described method (Rodriguez-Villodres et al., 2020). The 15 strains included 7 isolates with reduced susceptibility (MIC ≥ 4/4 μg/ml) and 8 randomly selected susceptible isolates (see above). In brief, *in vitro* selection was performed by inoculation of ∼10^8^ cfu in 2-ml LB broth containing CAZ-AVI at the 0.5 × MICs followed by incubation for 24 h. This procedure was repeated daily, each time doubling the CAZ-AVI concentration up to a maximum of 8/4 μg/ml. Selected colonies collected at different *in vitro* selection antibiotic levels were subject to CAZ-AVI susceptibility testing. The isolates with increased MICs ≥ 16/4 μg/ml after CAZ-AVI selection were determined as selected CAZ-AVI resistance.

## Results

### Distribution of the KPC-KP

The 616 non-duplicate clinical *K. pneumoniae* collected from September 2016 to June 2018 were from Chengdu (226, 36.7%, Southwest China), Kunming (148, 24.0%, Southwest China), Guangzhou (90, 14.6%, Southern China), Yinchuan (62, 10.1%, Northwest China), Suzhou (47, 7.6%, Eastern China), and Beijing (43, 7.0%, Northern China). In this collection 347 (56.3%) *K. pneumoniae* contained *bla*_*KPC*_, without coexistence of metallo-β-lactamases genes, the majority of which were from Kunming (119, 34.3%) followed by Chengdu (97, 28.0%), Guangzhou (49, 14.1%), Beijing (34, 9.8%), Suzhou (25, 7.2%), and Yinchuan (23, 6.6%) (Table 1).

**TABLE 1:** Distribution of the regions and ceftazidime-avibactam MICs of the KPC-KP.

**Regions**	**No. (%) of *K. pneumoniae***	**No. (%) of KPC-KP**	**No. (cumulative %) of KPC-KP inhibited at ceftazidime-avibactam MIC (μg/ml)**								**MIC_50_ (μg/ml)**	**MIC_90_ (μg/ml)**
			**≤0.125/4**	**0.25/4**	**0.5/4**	**1/4**	**2/4**	**4/4**	**8/4**	**16/4**		
Beijing	43 (7.0)	34 (79.1)	–	3 (8.8)	4 (20.6)	16 (67.6)	5 (82.4)	3 (91.2)	3 (100.0)	–	1/4	2/4
Chengdu	226 (36.7)	97 (42.9)	2 (2.1)	6 (8.2)	10 (18.6)	38 (57.7)	41 (100)	–	–	–	1/4	2/4
Guangzhou	90 (14.6)	49 (54.4)	–	4 (8.2)	2 (12.2)	20 (53.1)	21 (95.9)	2 (100.0)	–		1/4	2/4
Kunming	148 (24.0)	119 (80.4)	3 (2.5)	1 (3.4)	6 (8.4)	68 (65.5)	38 (97.5)	–	2 (99.2)	1 (100.0)	1/4	2/4
Yinchuan	62 (10.1)	23 (37.1)	–	1 (4.3)	4 (21.7)	6 (47.8)	12 (100.0)	–	–	–	2/4	2/4
Suzhou	47 (7.6)	25 (53.2)	1 (4.0)	1 (8.0)	4 (24.0)	13 (76.0)	5 (96.0)	1 (100.0)	–	–	1/4	2/4
**Total**	616 (100.0)	347 (56.3)	6 (1.7)	16 (6.3)	30 (15.0)	161 (61.4)	122 (96.5)	6 (98.3)	5 (99.7)	1 (100.0)	1/4	2/4

*–, no isolates.*

### Antimicrobial Susceptibility of KPC-KP

Minimal inhibitory concentrations of CAZ-AVI inhibiting KPC-KP ranged from ≤0.125/4 to 16/4 μg/ml, with only one strain which was resistant (16/4 μg/ml) according to the CLSI breakpoint (Clinical and Laboratory Standards Institute, 2018). The MIC_50_ was 1/4 μg/ml and MIC_90_ was 2/4 μg/ml (Table 1). We then used CAZ-AVI MIC of ≥4/4 μg/ml as the cut-off of reduced susceptibility to CAZ-AVI for this study (Shen et al., 2017). A total of 12 KPC-KP were found to have a CAZ-AVI MIC value ≥ 4/4 μg/ml with high level resistance to carbapenems (meropenem MICs ≥ 256 μg/ml) (Table 1).

These 12 isolates showed resistance to nearly all tested antimicrobials except trimethoprim-sulfamethoxazole (41.7%), chloramphenicol (16.7%), colistin (0%), and tetracycline (41.7%) (Table 2).

**TABLE 2:** Susceptibility of the 12 isolates against different antimicrobial agents.

**Antimicrobial agent^a^**	**Resistance^b^ ([n] %)**	**MIC (μg/ml)**	
		**50%**	**90%**
IPM	12 (100)	>8	>8
MEM	12 (100)	>8	>8
PIP	12 (100)	>64	>64
AMC	12 (100)	>16/8	>16/8
SAM	12 (100)	>16/8	>16/8
TZP	12 (100)	>64/4	>64/4
CZO	12 (100)	>16	>16
CTX	12 (100)	>32	>32
CAZ	12 (100)	>16	>16
FEP	12 (100)	>16	>16
ATM	12 (100)	>16	>16
GEN	12 (100)	>8	>8
AMK	12 (100)	>32	>32
CIP	12 (100)	>2	>2
MXF	12 (100)	>4	>4
lEV	12 (100)	>8	>8
SXT	5 (41.7)	≤0.5/9	>2/38
C	2 (16.7)	≤4	>16
CL	0 (0)	≤0.5	≤0.5
TET	5 (41.7)	8	>8

*^*a*^IPM, imipenem; MEM, meropenem; PIP, piperacillin; AMC, amoxicillin-clavulanate; SAM, ampicillin-sulbactam; TZP, piperacillin-tazobactam, CZO, cefazolin; CTX, cefotaxime; CAZ, ceftazidime; FEP, cefepime; ATM, aztreonam; GEN, gentamicin; AMK, amikacin; CIP, ciprofloxacin; MXF, moxifloxacin; LEV, levofloxacin; SXT, trimethoprim-sulfamethoxazole; C, chloramphenicol; CL, colistin; TET, tetracycline. ^*b*^ CLSI M100-S28 interpretive criteria were used in all cases, with the exception of MXF and CL, for which EUCAST breakpoints were used.*

### Clinical Information

The 12 isolates were obtained from distinct patients aged between 0 and 65 years old. They were from Beijing (*n* = 6), Kunming (*n* = 3), Guangzhou (*n* = 2), and Suzhou (*n* = 1). Four out of six Beijing isolates were collected from patients in ICU wards within the same month. In addition, most of these patients received empirical antibiotic treatments before these isolation of strains. Among them, carbapenems were the most commonly used antibiotic, followed by piperacillin-tazobactam (Table 3).

**TABLE 3:** Clinical information of the 12 patients connected with the isolates with reduced susceptibility to ceftazidime-avibactam.

**No.**	**City**	**Age range (y)**	**Isolated date**	**Clinical department**	**Source**	**Antibiotic treatment before isolated**	**Carbapenem usage (days)**	**Outcome**
1758	Beijing	55–60	Mar. 2017	ICU	Respiratory tract	Carbapenems	Meropenem (3)	Deterioration
1762	Beijing	30–35	Mar. 2017	ICU	Blood	Carbapenems	Meropenem (5) + Imipenem (5)	Improve
1764	Beijing	35–40	Mar. 2017	ICU	Intra-abdominal	Piperacillin-tazobactam, cephalosporin	–	Improve
1768	Beijing	45–50	Mar. 2017	ICU	Respiratory tract	Carbapenems, piperacillin-tazobactam, tigecycline	Meropenem (11)	Improve
2321	Beijing	35–40	Apr. 2017	General ward	Blood	Carbapenems	Imipenem (5)	Cure
2322	Beijing	35–40	Apr. 2017	General ward	Respiratory tract	Carbapenems	Imipenem (5)	Cure
2477	Kunming	0–1	Sep. 2017	General ward	Urinary tract	Fluoroquinolone	–	Improve
2789	Kunming	0–1	Oct. 2017	General ward	Urinary tract	Piperacillin-tazobactam	–	Improve
2827	Kunming	0–1	Nov. 2017	General ward	Urinary tract	Piperacillin-tazobactam	–	Deterioration
1706	Guangzhou	60–65	Feb. 2017	ICU	Blood	Carbapenems, piperacillin-tazobactam, vancomycin	Meropenem (7) + Imipenem (1)	Improve
5152	Guangzhou	55–60	Sep. 2018	Organ transplantation department	Blood	Not clear	–	Not clear
3855	Suzhou	45–50	Apr. 2018	General ward	Intra-abdominal	Fluoroquinolone	–	Improve

*“–”, no carbapenems was used.*

### Detection of ESBL and Virulence Genes and Capsular Genotyping

These 12 isolates contain ESBLs genes including *bla*_*CTX–M–65*_ (*n* = 9), *bla*_*CTX–M–14*_ (*n* = 3) and *bla*_*SHV–12*_ (*n* = 8). In addition, the results of multiplex PCR showed that 6 isolates from Beijing were positive for the four virulence genes, namely *rmpA*, *rmpA2*, *iroN*, and *iutA*. Eight isolates belonged to capsular type KL64 and the other 4 isolates were from KL47 (Table 4).

**TABLE 4:** Molecular characteristics of the 12 KPC-KP with reduced susceptibility to ceftazidime-avibactam.

**No.**	**β-lactamases genes**	**Virulence genes**	**Sequence type**	**Capsular type**	**OmpK35**	**OmpK36**
1758	*bla*_*KPC–2*_, *bla*_*CTX–M–65*_, *bla*_*SHV–12*_	*rmpA*, *rmpA2*,*iutA*, *iroN*	ST11	KL64	Stop codon	134-135 GD
1762	*bla*_*KPC–2*_, *bla*_*CTX*_*_–M–65_, bla*_*SHV–12*_	*rmpA*, *rmpA2*,*iutA*, *iroN*	ST 11	KL64	Stop codon	134-135 GD
1764	*bla*_*KPC–2*_, *bla*_*CTX–M–65*_, *bla*_*SHV–12*_	*rmpA*, *rmpA2*,*iutA*, *iroN*	ST 11	KL64	Stop codon	134-135 GD
1768	*bla*_*KPC–2*_, *bla*_*CTX*_*_–M–65_*	*rmpA*, *rmpA2*,*iutA*, *iroN*	ST 11	KL64	Stop codon	134-135 GD
2321	*bla*_*KPC–2*_, *bla*_*CTX–M–65*_, *bla*_*SHV–12*_	*rmpA*, *rmpA2*,*iutA*, *iroN*	ST 11	KL64	Stop codon	134-135 GD
2322	*bla*_*KPC–2*_, *bla*_*CTX–M–65*_, *bla*_*SHV–12*_	*rmpA*, *rmpA2*,*iutA*, *iroN*	ST 11	KL64	Stop codon	134-135 GD
2477	*bla*_*KPC–2*_, *bla*_*CTX–M–14*_, *bla*_*SHV–12*_	–	ST 11	KL47	Stop codon	134-135 GD
2789	*bla*_*KPC–2*_, *bla*_*CTX–M–14*_	–	ST 11	KL47	Stop codon	134-135 GD
2827	*bla*_*KPC–2*_, *bla*_*CTX–M–14*_, *bla*_*SHV–12*_	–	ST 11	KL47	Stop codon	134-135 GD
1706	*bla*_*KPC–2*_, *bla*_*CTX–M–65*_, *bla*_*SHV–12*_	–	ST 11	KL64	Stop codon	134-135 GD
5152	*bla*_*KPC–2*_, *bla*_*CTX–M–65*_	–	ST 11	KL47	Stop codon	134-135 GD
3855	*bla*_*KPC–2*_, *bla*_*CTX–M–65*_	–	ST 11	KL64	Stop codon	134-135 GD

*“–”, negative for PCR detection.*

### MLST Sequence Types and PFGE Patterns

**FIGURE 1:**
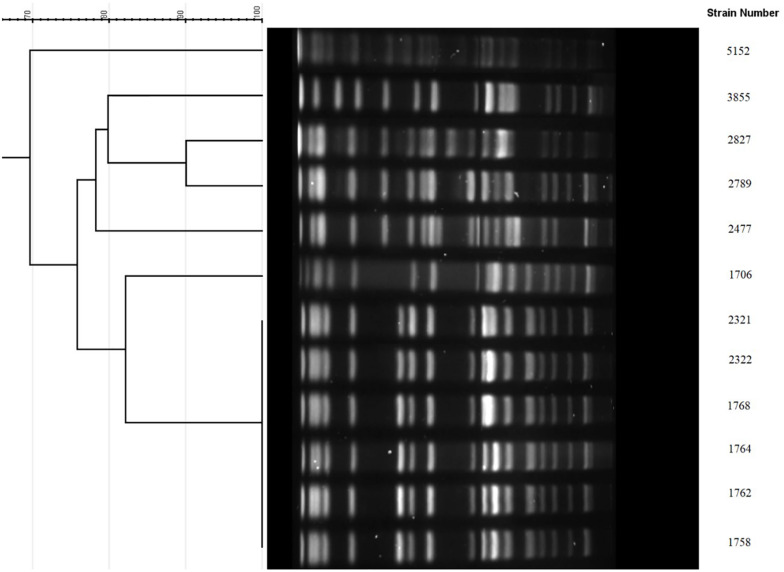
PFGE cluster analysis of the 12 KPC-KP with reduced susceptibility to ceftazidime-avibactam. 1758, 1762, 1764, 1768, 2321, and 2322 were from Beijing sharing the same PFGE pattern. 2477, 2789, and 2827 were from Kunming, among which 2789 and 2827 were highly homologous (>90%). 1706 and 5152 were from Guangzhou. 3855 was from Suzhou.

Multilocus sequence typing results showed that all 12 isolates belonged to ST11. Furthermore, these 12 isolates were investigated by PFGE. As shown in Figure 1, the 6 isolates from Beijing shared the same PFGE pattern. In addition, 2 of the 3 isolates from Kunming were highly homologous (>90%, dice similarity coefficient). The other isolates demonstrated different pulsotypes.

### Outer Membrance Porin Gene Sequence Analysis

Sequencing of the outer membrane porin genes *ompK35* and *ompK36* showed that all 12 isolates contain a mutant OmpK35, with a premature stop codon at amino acid position 63, as well as a mutant OmpK36, due to the glycine and aspartic acid duplication at amino acid 134 (134–135 GD insertion). However, the OmpK35 and OmpK36 gene sequences of 8 randomly selected susceptible *K. pneumoniae* ST11 isolates showed the same genotypes (OmpK35 stop codon and OmpK36 134–135 GD insertion) as those of the 12 isolates with reduced CAZ/AVI susceptibility.

### Mechanism of Reduced CAZ-AVI Susceptibility

**FIGURE 2:**
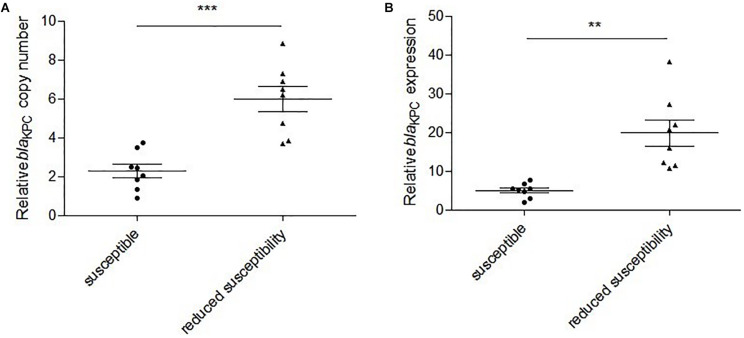
Relative *bla*_*KPC–2*_ copy numbers and expressions in susceptible and reduced susceptibility isolates. **(A)** Relative *bla*_*KPC–2*_ copy numbers in selected isolates. ***P = 0.0004, Unpaired *t* test with Welch’s correction. **(B)** Relative *bla*_*KPC–2*_ expressions in selected isolates. **P = 0.0034, Unpaired *t* test with Welch’s correction.

Polymerase chain reaction detection and Sanger sequencing showed that the 12 isolates all harbored wild-type *bla*_*KPC–2*_. Examination of the *bla*_*KPC–2*_ promoter regions failed to identify any mutations in comparison to the sequences from the susceptible strains. The results of qRT-PCR showed that the relative *bla*_*KPC–2*_ copy numbers in the reduced susceptibility group were significantly higher than those in the susceptibility group (2.6-fold, *P* = 0.0004) (Figure 2A). In addition, the relative expressions of *bla*_*KPC–2*_ in the reduced susceptibility group were 3.9-fold higher than those in the susceptibility group (*P* = 0.0034) (Figure 2B).

### *In vitro* Selection Testing

The *in vitro* selection experiments showed that 4 of the 7 isolates with reduced susceptibility developed resistance to CAZ-AVI at the selection concentration of 2/4 μg/ml, and all 7 isolates developed resistance to CAZ-AVI with MICs ranging from 32/4 to 256/4 μg/ml when the selection concentration reached 4/4 μg/ml. By contrast, no isolates were developed to be resistance to CAZ-AVI in the susceptibility group, and all eight isolates stopped growing when the selection concentration reached 8/4 μg/ml.

## Discussion

At present time, KPCs are the most common carbapenemases identified worldwide especially in *K. pneumoniae* from China (Nordmann and Poirel, 2014). Due to the limited therapies, the detection rate of KPC-KP in China demonstrated a continuous upward trend (Hu et al., 2016). In this study, CAZ-AVI showed potent *in vitro* activities against KPC-KP, which agreed with previous studies (de Jonge et al., 2016; Spiliopoulou et al., 2020). However, ∼3% isolates displayed reduced susceptibility to CAZ-AVI (MIC ≥ 4/4 μg/ml) despite they were obtained from patients without previous CAZ-AVI treatment history. Notably, these isolates also showed high level resistance to carbapenems.

In 2018, a fatal outbreak caused by ST11 KPC-KP with acquisition of a pLVPK-like virulence plasmid that increased the virulence of these isolates was reported (Gu et al., 2018). In our study, half of the isolates with reduced CAZ-AVI susceptibility contained several known virulence genes, suggesting that these isolates may have increased virulence, which should be closely monitored.

Since the previously reported gene mutations (e.g., D179Y) associated to CAZ-AVI resistance (Giddins et al., 2018) weren’t found in the *bla*_*KPC–2*_ genes, this study suggested that mechanisms other than *bla*_*KPC–2*_ gene mutation were underlying the reduced CAZ-AVI susceptibility among these isolates. OmpK35/36 defects had previously been reported to lead to reduced susceptibility or resistance to CAZ-AVI in KPC-KP (Nelson et al., 2017). In this study, our results showed 12 isolates with reduced CAZ-AVI susceptibility contained OmpK35 and OmpK36 gene mutations, however, the same gene mutations were also found in the susceptible strains, which suggested that OmpK35/36 defects may only partially contribute to the reduced CAZ-AVI susceptibility among those 12 strains, while additional mechanisms may be involved. Our results demonstrated that the *bla*_*KPC–2*_ copy numbers and expressions in the reduced susceptibility group were significantly higher than those in the susceptibility group. We therefore suspected that the reduced CAZ-AVI susceptibility in these strains was likely due to the higher *bla*_*KPC–2*_ copy numbers and gene expressions, in combination to the OmpK35/36 defects. The results were consistent with some previously published studies (Shen et al., 2017; Zhang et al., 2020). The higher copy numbers of *bla*_*KPC–2*_ may potentially result from the high copy numbers of the plasmids carrying *bla*_*KPC*_ (Roth et al., 2011) and/or a duplication of mobile genetic elements associated with *bla*_*KPC*_ (Coppi et al., 2020). Since the examination of the *bla*_*KPC–2*_ promoter regions failed to identify any mutations, the higher *bla*_*KPC*_ gene expression may potentially be affected by the higher copy numbers of *bla*_*KPC*_ or other gene regulatory mechanisms. Further studies, including whole genome sequences, are needed to explore the molecular mechanisms underlying the higher *bla*_*KPC–2*_ copy numbers and gene expressions among those strains.

Shields et al. (2017) has reported on the acquisition of CAZ-AVI resistance among ST258 KPC-KP during treatment in the United States. After that, (Raisanen et al., 2019) isolated a ST39 KPC-KP that was resistant to CAZ-AVI after this combination treatment. In China, ST11 KPC-KP is commonly prevalent (Chen et al., 2014; Zhang et al., 2017). In this study, the ST11 KPC-KP with reduced susceptibility were more prone to develop CAZ-AVI resistance compared to susceptible isolates under the pressure of CAZ-AVI exposing.

Taken together, our study demonstrated that CAZ-AVI has potent *in vitro* activities against KPC-KP in China and highlighted the clinical significance of the isolates with reduced susceptibility to CAZ-AVI isolated from patients without previous CAZ-AVI treatment history. Our results suggested that the optimal clinical usage of CAZ-AVI should be guided by the *in vitro* susceptibility results in order to prevent selection resistance.

## Data Availability Statement

The datasets generated for this study are available on request to the corresponding author.

## Ethics Statement

This study was approved by the institutional review board (IRB) of The Second Affiliated Hospital of Soochow University. The clinical isolates were retrospectively collected, and patient data were not included in this study, therefore the need for written informed consent was waived by the IRB.

## Author Contributions

XC, BS, and XZ contributed to conducing the study, data analysis, and manuscript preparation. FQ, WJ, BH, HY, and Y-WT analyzed the data and reviewed the manuscript. LC and HD contributed to study design, data analysis, and manuscript preparation. All authors contributed to the article and approved the submitted version.

## Conflict of Interest

Y-WT was employed by Cepheid, Shanghai, China. The remaining authors declare that the research was conducted in the absence of any commercial or financial relationships that could be construed as a potential conflict of interest.

## Footnotes

1 https://bigsdb.pasteur.fr/klebsiella/klebsiella.html
